# Chemical Composition of Newborn Piglets with Different Weights at Birth in Sows with a High Reproductive Performance

**DOI:** 10.3390/ani14091380

**Published:** 2024-05-04

**Authors:** Carina Antonczyk, Christine Ratert, Cornelia Schwennen, Josef Kamphues, Amr Abd El-Wahab

**Affiliations:** 1 Institute for Animal Nutrition, University of Veterinary Medicine Hannover, Foundation, Bischofsholer Damm 15, D-30173 Hannover, Germanycornelia.schwennen@tiho-hannover.de (C.S.); josef.kamphues.ir@tiho-hannover.de (J.K.); 2 Department of Nutrition and Nutritional Deficiency Diseases, Faculty of Veterinary Medicine, Mansoura University, Mansoura 35516, Egypt

**Keywords:** piglets, body composition, birth weight, placenta

## Abstract

**Simple Summary:**

Within recent decades, the reproductive performance of sows has risen enormously thanks to efforts in breeding, i.e., litter sizes with more than 15–16 piglets. These changes in litter size have had considerable effects on the individual birth weights of piglets, as well as on the variation in birth weights within a litter (e.g., proportion of “underweight” newborns). The present study showed that the energy and protein accumulation in the fetus and placenta increased by about 75% and 64%, respectively, in comparison to studies as far as 30 years ago, in which the litter size varied around 10–12 piglets. Also, the newborn piglets with a body weight of 1.0 kg scarcely have impaired chances of survival, which is reflected in their normal energy content. On the other hand, the energy and nutrient supply for high yielding sows at the end of gestation must be significantly augmented, as the main influencing factors, namely energy and protein accretion, have increased considerably due to the marked higher masses of fetuses.

**Abstract:**

The present study aimed to quantify and update the data on the body composition (energy nutrients) of newborn piglets of different body weights at the time of birth, as well as of the placenta mass. Data were collected from newborn piglets (*n* = 25) from modern genetic lines which were stillborn or died within the first 24 h of life after being crushed to death with various body weights at birth (<0.8 kg (*n* = 5); 0.8–1.2 kg (*n* = 5); >1.2–1.6 kg (common birth weight, *n* = 10) and >1.6 kg (*n* = 5)). The placenta (*n* = 20) of sows from a conventional breeding farm were collected, too. The body composition of newborns of “normal” (>1.2–1.6 kg) and even lighter (0.8–1.2) weights still indicated a “normal” composition. In the case of a lower body weight of piglets <0.8 kg at birth, the crude ash (24.1%) and crude protein (8.21%) contents were higher, but the crude fat (16.1%), carbohydrate (57.4%), and gross energy (3.60%) contents were lower. The placental composition in comparison to the piglet body composition was characterized by higher crude protein contents (24.3%) and lower crude ash (31.6%), crude fat (9.08%), and carbohydrate (55.6%) contents. In conclusion, the energy and protein accumulation in the total mass of fetuses and placentas increased by 75% and 64%, respectively, in comparison to times in which the litter size varied around 10–12 piglets, essentially as a result of the larger fetal mass and not of a different body composition.

## 1. Introduction

The productivity of modern high reproductive sows has steadily increased in recent years. To improve the efficiency of sow husbandry, there has been a strong selection over the past three decades for highly prolific sows. These efforts have resulted in higher litter sizes and, from an economic point of view, have improved productivity [1,2]. However, this increase in litter size is accompanied by a higher variation in birth weight and a higher share of piglets with an abnormally low birth weight (BW) [3,4]. In a study by [3], it was shown that, with an increase in the litter size of piglets born from ≤11 to ≥16 (dead and alive), the absolute litter size increased by 88% (9 vs. 17 total piglets born), but the litter mass only increased by 50% (from 14 to 21 kg), resulting in a reduced mean birth weight, ranging from 1.59 to 1.26 kg.

Piglet viability is a major challenge in the swine industry, particularly in recent years, due to the marked increase in prolificacy. In commercial farming, it is estimated that 15–20% of piglets are lost during the first days of life (mainly as a result of crushing, starvation, and chilling) [5]. This is a matter of growing importance in the swine industry because it not only impairs productivity, but also seriously compromises animal welfare and the social acceptability of farming practices. The birth weight of piglets was the most determining factor on the final weight at the end of the nursing period. On the other hand, the sex of the piglets showed no significant influence on the final weight [6].

Neonatal death is particularly concentrated in low birth weight (LBW) piglets, which is usually associated with limited energy reserves, hypothermia, anoxia, and low vitality and mobility during the initial hours [7]. The proportion of LBW piglets (<1.1 kg) in litters with more than 15 piglets is above 15–20% [8,9,10]. However, the lack of a consistent across-studies criterion to identify the targeted LBW piglets limits the comparison of results between studies and obscures drawing a conclusion on the effect of these compounds [11,12,13]. While some authors apply birth weight cut-offs up to 1.00–1.35 kg to define LBW piglets [14,15,16], others use lower birth weights [17,18,19].

Several strategies have been proposed to reduce neonatal mortality, which involve all aspects of swine production, including nutritional strategies to improve fetal development, management practices during gestation, and transitional and peripartum periods to improve the survival rate and performance of neonatal piglets [5,20]. This combination of strategies has been reported to reduce neonatal mortality, and a figure of 5–7% may be considered achievable [21]. Nevertheless, the implementation of these management practices within production is usually low, as it requires extra labor costs, which, in many cases, do not compensate the potential benefit of reducing neonatal mortality. On the other hand, low BW is associated with different developmental patterns and changes in body composition and homeostasis, due to prenatal programming through epigenetic changes [22,23,24], which affects the profitability and production of commercial pig products [25].

Against this background, two fundamental questions arise from the viewpoint of animal nutrition: firstly, the question regarding the body composition of the individual piglets, which in fact also determines their chances to survive. Secondly, the question regarding the consequences for the energy and nutrient supply for sows during late gestation. Do they only produce a higher fetal mass or, in fact, piglets with a different body composition? Therefore, the present study aimed to quantify and update values on the energy and nutrient contents of newborn piglets of different body weights at the time of birth, as well as on the mass and composition of placenta, assuming that both are changing, too.

## 2. Materials and Methods

All the animals were housed in accordance with the EU (European Directive 2008/120/EU) [26]. In compliance with European Directive 2010/63/EU, the present study did not imply any invasive procedure or treatment to the animals. The study was reviewed and approved by the Animal Welfare Committee of the University of Veterinary Medicine Hannover, Foundation, Germany (TVO-2016-V-32).

### 2.1. Farms, Piglets, and Placenta

Three German sow farms with different genetics (JSR Hybrid, Bundes Hybrid Zucht Programm (BHZP)) have been engaged in our study. The piglets were both male and female, but the sexes were not recorded individually. For the body composition analyses, 25 newborn piglets of the JSR Hybrid and BHZP lines were divided into four groups depending on their BW at birth (<0.8 kg (*n* = 5); 0.8–1.2 kg (*n* = 5); >1.2–1.6 kg (*n* = 10); >1.6 kg (*n* = 5)). The selected piglets were either stillborn or died within the first 24 h of life due to weakness or being crushed by the sows. Moreover, placentas (BHZP line) were collected from sows from different farms. For this purpose, tarpaulins were placed behind the animals to prevent the placenta from slipping through the slatted floor and to ensure that the resulting material could be collected cleanly and completely. The total placentas of 20 sows were initially weighed as a whole. The total masses ranged from 3.0 to 7.2 kg. In 12 litters, the total weight of all newborns (piglets born alive and dead, i.e., the whole litter) was recorded in addition to the placental weight.

### 2.2. Sample Preparation

The piglets were first cut into approximately fist-sized pieces in their frozen state using a band saw, and were then minced in a conventional meat cutter. At the same time, the material was thoroughly mixed by hand. The chopped material was first freeze-dried for at least three days until it was brittle and could be smashed using a rubber mallet, so that approximately 3 g of the sample material could be removed for hot drying. The material was then further crushed in a Grindomix (GM 200, Retsch GmbH, Haan, Germany) at 8500 rpm for seven seconds. A compaction lid was also used to reduce the size of the grinding chamber. The finest degree of comminution was achieved via subsequent dry grinding in the vibrating mill (MM 400, Retsch GmbH). A very fine particle size of the sample is essential in order to be able to take a representative sample from the original total amount of material and make it available for the subsequent chemical analyses.

A similar procedure was followed with the placenta, which were first homogenized in a blender (Blendor 800E, Waring Products Division Dynamics Corporation of America, New Hartford, CT, USA). Approximately 100 g of the sample material was taken from the pre-crushed material to determine the dry matter content, and an aliquot of approximately 1000 g was taken, which was freeze-dried and then ground in a Grindomix (GM 200, Retsch GmbH). This was followed by grinding using a vibrating mill (MM 400, Retsch GmbH) in order to achieve the highest possible degree of comminution.

### 2.3. Chemical Analysis

The chemical analysis of all samples was carried out in duplicate. To determine the nutrients in the samples, the procedures from the Association of German Agricultural Analytical and Research Institutes e.V. [27] were used. The dry matter (DM) content was calculated by weighing the samples (about 50 g) before and after they had been dried at 103 °C for 12 h. The crude ash content was determined by weighing the dried and ground samples (about 3 g) before and after combustion at 600 °C for 6 h in the muffle furnace. The Dumas incineration method was also used to determine the total nitrogen content by heating about 0.3 g of the sample in a crucible in the elemental analyzer (VarioMax^®^ CNS, Fa. Elementar Analysesysteme GmbH, Langenselbold, Germany). The crude protein could then be calculated via multiplying the nitrogen content by 6.25. The crude fat was then extracted over 6 h in a Soxhlet apparatus using a petroleum ether. After drying the flask used for this until a constant weight is reached, the petroleum ether was distilled off again using a rotary evaporator (Hei-VAP Value, Heidolph Instruments GmbH & Co. KG, Schwabach, Germany). The flask was then dried again overnight (80 °C) and reweighed after cooling in the desiccator. The nitrogen-free extract (NfE) was calculated using the following formula: NfE = DM − (crude ash + crude protein + crude fat) as the crude fiber can not occur or be determined.

The calcium content was determined using atomic absorption spectrometry (Unicam SOLAAR M Series Flame and Furnace Atomic Absorption Spectrometer Systems, Fa. Thermo Elemental/Thermo Scientific, Cambridge, UK), in accordance with (Slavin, 1982). A photometric characterization (UV–Visible Recording Spectrophotometer UV 162, Schimadzu, Kyoto, Japan; Wavelength 356 nm) of the phosphorus content was based on the vanadate-molybdate method, in accordance with Gericke and Kurmies [28].

In brief, to determine the chloride content, about 5 g of the sample material required for this was weighed into a 50 mL volumetric flask, which was filled with approx. 35 to 40 mL of distilled water and then shaken for 30 min on a shaking plate. The solution was then made up to a volume of 50 mL with distilled water, and was then centrifuged (15 min, 3000 rpm) so that the chloride content could be determined in the following step using precipitation titers in the Chloride Analyzer 925 (Ciba Corning Diagnostics, Medfield, MA, USA).

To estimate the amount of selenium, the sample solution was prepared using wet ashing. For this purpose, 1 g of the ground sample was mixed with 15 mL of an ashing mixture, consisting of perchloric acid (70%) and nitric acid (65%), and carefully heated. After the sample has been stirred, the sample was boiled twice with 5 mL HCl (7.5%) and then left to stand until cooled. After adding a further 10 mL of semi-concentrated HCl and heating it in a water bath for 30 min, the filtration of the sample was performed. The treatment process produced selenium hydride, which was then measured using a hydride system (VP 90, Unicam, Cambridge, UK) coupled with an Atomic Absorption Spectrometer (Solar 969, Unicam, Cambridge, UK). Finally, the amino acid contents were determined using ion-exchange chromatography (AA analyzer LC 3000, Laborservice Onken GmbH, Gründau, Germany).

The gross energy (GE) content in MJ/kg DM could be calculated using the following formula [29]: GE (MJ/kg DM) = 0.0239 × crude protein + 0.0398 × crude fat + 0.0175 × nitrogen-free extract. In addition, the calculated derivation was checked via gross energy determination in the Bomb Calorimeter (C2000 basic calorimeter system, IKA-Werke, Staufen, Germany) at the Institute of Physiology, Chair of Animal Nutrition and Dietetics, Ludwig Maximilian University of Munich, Germany. For the analyses, 0.5 g of the finely ground sample material was pressed into tablets using a briquetting press (C21, IKA Werke, Staufen, Germany) and were then incinerated in a five-fold process.

### 2.4. Statistical Analyses

Statistical tests were performed using the Statistical Analysis System for Windows, version SAS^®^ 9.3 (SAS Institute Inc., Cary, NC, USA). The data were compared in the case of normal distribution using 1-factorial analysis of variance, and, in the case of non-normally distributed data, using non-parametric methods (Kruskal–Wallis test or Wilcoxon two-sample test). For example, the data of chemical composition of newborn piglets were statistically analyzed according to weight classes (<0.8; 0.8–1.2; >1.2–1.6; >1.6 kg). Significant differences with a probability of error of *p* < 0.05 were labeled accordingly with different superscript letters.

## 3. Results

### 3.1. Chemical Body Composition of Piglets

The chemical body composition of the newborn piglets depending on the BW at the time of birth is presented in Table 1. The average crude ash content of piglets with a BW of <0.8 kg at the time of birth was significantly higher than that of piglets with a BW > 0.8 kg, and remained constant from a BW of >0.8 kg at the time of birth. Similarly, the very light piglets (<0.8 kg) had a significantly higher crude protein content (about 65% of DM) than the piglets in the other three weight classes (about 60% of DM). The results of the amino acid contents are shown as a Supplementary File (Table S1); however, due to the number of investigations being low (*n* = 2), the data of the amino acid contents can not be accurately and/or statistically presented.

Piglets with a BW of <0.8 kg at the time of birth also had significantly lower crude fat levels (60.1 g/kg DM) when compared to piglets with >0.8 kg BW. From a BW of >0.8 kg at the time of birth, this was relatively constant, at approx. 70 g/kg DM. The piglets with less than 0.8 kg BW at the time of birth had significantly lower GE contents than newborns with a BW > 0.8 kg. The GE content of piglets from the weight classes 0.8–1.2 kg, >1.2–1.6 kg, and >1.6 kg did not differ significantly from each other.

### 3.2. Nutrient and Energy Contents of Piglets (Stillborn or Liveborn)

Stillborn individuals (*n* = 5) were allocated to the weight classes > 1.2–1.6 kg BW (*n* = 3) and >1.6 kg BW (*n* = 2). Thus, the stillborn piglets in the weight class > 1.2–1.6 kg DM were compared with the mean value of the liveborn piglets from their weight class (Table 2). No significant differences were noted in the nutrient and energy contents of stillborn or liveborn piglets (> 1.2–1.6 kg BW) at the time of birth.

### 3.3. Mineral Content of Newborn Piglets

The mineral content of newborn piglets according to their body weight at birth is shown in Table 3. Newborn piglets of <0.8 kg BW had significantly the highest content of major minerals when compared to other BW classes (*p* < 0.05), whereas no significant differences were noted in the levels of major minerals between different BW groups (0.8–1.2, >1.2–1.6, >1.6 kg). Regarding the trace elements, only the zinc level showed significant differences among the groups. The BW groups of >1.2–1.6 kg and >1.6 kg had significantly the lowest zinc levels when compared to other BW groups. The data of the iron contents were 136, 135, 173, and 180 mg/kg DM for piglets of <0.8, 0.8–1.2, >1.2–1.6, >1.6 kg, respectively (no statistical analyses were done as the numbers (n) of the analyses were very low).

### 3.4. Nutrient and Energy Content of Placenta

The placental weight (kg, mean) was about 3.93 ± 0.570, while the number of total piglets born (either alive or dead) was 14.6 ± 2.54 and the litter mass (kg) of 12 litters was 20.6 ± 2.17. Regarding the placental weight (g fresh basis) per kg of piglet weight, this amounted to about 191 ± 19.1, whereas the placental weight (g DM basis) per kg of piglet weight was 14.3 ± 1.07.

The nutrient content of placenta is presented in Table 4. The level of crude ash varied at 128 g/kg DM, while the crude protein content was 742 g/kg DM. The levels of both crude fat and NfE were comparable to each other, namely 64–65 g/kg DM. The contents of sodium and chloride were a great deal higher (32.3 and 42.1 g/kg DM, respectively) when compared to other major elements, whereas the level of magnesium was the lowest (1.07 g/kg DM). The levels of trace minerals varied greatly, while the iron content was the highest (367 mg/kg DM) and the level of selenium was the lowest (1.23 mg/kg DM).

## 4. Discussion

Some of the piglets that died within the first 24 h of life or were stillborn were collected by the authors as well as the workers on the farms. Due to the injuries and/or alterations in the carcass after death by crushing, it can be assumed that the piglets that died due to crushing by the sow and the piglets that died due to weakness could be easily distinguished. It proved more difficult to accurately differentiate between stillborn piglets and piglets that died shortly after birth, as no lung float test was carried out on the piglets and the farm workers were not present at the births that took place overnight. Each piglet used in the study was weighed after death and were then stored frozen. As some piglets were kept for longer, for example, by the farm owners, they were already deep-frozen at the time the BW was recorded. The freezing process may result in slight deviations from the actual BW at the time of birth due to moisture loss. The samples of placenta were collected exclusively by the authors and under clean conditions, so that no contamination (e.g., by bedding materials, feces, slatted floors) was to be expected during the collection phase. However, the amount of amniotic and allantoic fluids present may vary, which might have influenced the DM content of the placenta. The amount of blood adhering to the placenta can also vary, which may influence both the DM content and the chemical composition (for example, sodium and iron).

The results of the present study regarding the DM content of newborn piglets are consistent with those of previous studies; the DM content varied between 18 and 20% [30,31,32]. According to the literature, the water content of newborn piglets varies between 750 and 850 g/kg of total BW, regardless of the BW of the animals [30]. This can be confirmed by the present results, and is also consistent with the results of Sagel [33], who also performed the chemical analyses of newborn piglets considering the BW at the time of birth. In the present study, the lightest piglets had numerically lower DM contents than heavier ones, albeit not significantly. Pig fetal growth increases during the second half of gestation [34,35]. Several factors, including genetics, nutrition, and the gestational stage, are major issues of growth rates in fetal pigs [36,37,38].

The crude protein content (on a DM basis) of a newborn piglet is relatively high and, according to Becker [30], is also not subject to the influence of the BW at the time of birth. According to Becker [30], values of 100–150 g/kg of fresh weight basis are achieved. Those values fundamentally agree with the results of the present study, but the light piglets, <0.8 kg in BW, in particular achieved significantly higher crude protein contents based on DM at the time of birth. Therefore, there is definitely a dependency here in relation to the BW at the time of birth that was not alluded to by Becker [30]. The reduced feed intake might entail that a greater proportion of the ingested energy is retained as protein, but the issue might not be so straightforward if, as suggested by some studies, those pigs had an increased lysine catabolism [39,40]. Moreover, McPherson et al. [41] showed that a fetal protein accretion increased in a quadratic manner (*p* < 0.001) after d 69 of gestation in gilts when compared to before d 69 of gestation (4.63 g/d vs. 0.25 g/d), indicating that protein growth increased markedly after d 69 of gestation.

On the other hand, Sagel [33] observed slightly higher crude protein contents (on a fresh basis) in the heavy piglets. When compared to the results of Beyer et al. [31], who determined the crude protein contents in the range of 569 and 594 g/kg DM, those findings are consistent with the contents of piglets from the present study with a BW of >1.2 kg at the time of birth, but not with those of piglets <1.2 kg BW, as the crude protein content of the lighter piglets had numerically higher values.

The crude fat content of a newborn piglet was stated by Becker [30] to be 12–17 g/kg (on a fresh basis). These values appear quite high in comparison to the results of the present study, as only a few piglets (*n* = 6) in our study reached crude fat levels above 15 g/kg (on a fresh basis). Sagel [33] and Beyer et al. [31] also reported lower crude fat levels. Sagel [33] determined crude fat contents of 9.8 g/kg (fresh basis) in light piglets and 10.7 g/kg (on a fresh basis) in heavy piglets; the correlation calculated as a function of DM was 0.37. Our results agree with those of Beyer et al. [31], who reported crude fat contents of 9.18–11.6 g/kg (fresh basis) and 54–63 g/kg in DM. The crude fat contents (in DM) in the present study deviated slightly upwards. Only piglets with a BW of <0.8 kg at the time of birth had crude fat contents in the range mentioned by Beyer [31]; heavier piglets at the time of birth had higher crude fat contents of around 70 g/kg DM. This observation is very interesting against the background of breeding activities for a low-fat meat pig, as it would suggest lower crude fat contents at the time of birth. McPherson et al. [41] noted that, with the late stages of gestation in gilts, there was an increase in the fat level of fetuses in a quadratic manner (R2 = 0.904; *p* < 0.001). Moreover, McPherson et al. [41] found that, before day 69 of gestation, the fetal fat accretion was 0.06 g/day (*p* < 0.001), which increased to 1.1 g/day (*p* < 0.001) thereafter, indicating an acceleration in fat growth after day 69 of gilt gestation.

According to Becker [30], the crude ash content of a newborn piglet assumes values of between 35 and 40 g/kg (on a fresh basis) and always represents a larger fraction than the crude fat content in the newborn piglet. Sagel [33] determined crude ash contents of 41.8–43.5 g/kg (fresh basis) and found no significant differences depending on the BW of the piglets at the time of birth. In the present study, crude ash levels ranged from 34.6 (>1.6 kg BW) to 42.5 g/kg BW (<0.8 kg BW), and were thus significantly higher in BW and DM (185 g/kg DM in piglets > 1.6 kg BW; 232 g/kg DM in piglets <0.8 kg BW) in the light piglets than in the heavier piglets. When compared to the results of Beyer et al. [31] who determined the crude ash contents in the range of 163–185 g/kg DM, the light piglets in the present study with a BW of <0.8 kg had significantly higher crude ash contents, at 232 g/kg DM. This observation suggests a relatively higher skeletal content in very light piglets, possibly at the expense of crude fat and NfE contents. Moreover, Mahan et al. [42] illustrated that both the macro- and microminerals increased in a curvilinear fashion (*p* < 0.01) as the fetal development advanced, with roughly 50% of the total litter and fetal macro- and micromineral contents accumulating during the final 15 days of gestation. The study suggested a significant marked increase in the mineral contents in fetal pigs during late gestation, implying a possibly heightened mineral requirement for sows, especially those with larger litter sizes. Overall, as gestation progresses, the total fetal mineral demand is presumed to rise, potentially leading to the mobilization of body minerals in sows, particularly if dietary mineral provisions are insufficient.

The NfE content was calculated via subtracting the sum of the crude nutrients from the factor 1000; from this, it was obvious that this fraction would be low in the very light piglets, as they are already relatively rich in crude ash and crude protein. Consequently, piglets with a BW <0.8 kg at the time of birth also had significantly lower NfE contents than piglets with a BW > 0.8 kg. It can be assumed that the NfE fraction of the piglet mainly represents glycogen stored in the liver and muscle [43], which serves as a rapidly available energy source for the piglet in the first hours postnatum. Only by utilizing energy in the form of glycogen can the newborn maintain its body temperature and thus ensure its survival [44]. Unfortunately, these very light piglets have a higher energy requirement needed to maintain their body temperature per kg of BW at the time of birth [45], as they have a less favorable ratio of body surface area to body mass. Thus, at this point, the NfE fraction appears to be a first possible limiting factor for neonatal survival. The rapid consumption of carbohydrates postnatum was demonstrated by comparing the NfE content of live and stillborn piglets. When compared to previous studies, however, the NfE content of the newborns appears to have increased overall. While Sagel [33], for example, estimated 126 g NfE/kg DM for the 1.2–1.5 kg weight class, in the present study, the figure for the comparable weight class (1.2–1.6 kg DM) was 145 g NfE/kg DM. The heavy piglets also showed significant upward deviations (134 g NfE/kg DM according to Sagel [33], and 148 g NfE/kg DM in the present study).

The calculated GE content of the newborn piglet is determined by its crude protein, crude fat, and NfE content. Crude fat has the highest caloric value (36.6–39.8 KJ GE/g), followed by crude protein (23.9–24.2 KJ GE/g) and then the NfE fraction (17.0–17.5 KJ GE/g). However, since the survival of a newborn in the first few hours postnatum can be largely ensured by the available glycogen reserves, but these have a much lower impact on the GE calculation than the crude protein and crude fat contents due to their relatively low average calorific value, it can be concluded that the calculated GE content cannot serve as a measure of possible survival chances. If the GE content is calculated from the data on crude nutrients from the study by Sagel [33], the GE content of today’s newborns is generally higher (about 1 MJ/kg DM in the comparable BW classes). Whereas, in the study by Sagel [33], a GE content of 18.6 MJ/kg DM can be derived from the available data for piglets with a BW < 1.2 kg; piglets with a BW < 0.8 kg already had a calculated GE content of 18.9 MJ/kg DM in the present study. With the increasing number of very light piglets with increasing litter sizes, this plays a significant role for the sow’s overall requirement towards the end of the pregnancy, as the sow’s energy retention in the piglets could then also assume higher values. Previously, 2.5 MJ GE/day was assumed in the last trimester of pregnancy. If a further comparison is made between the weight classes chosen by Sagel [33] of 1.2–1.5 kg BW and >1.5 kg BW and the weight classes 1.2–1.6 and >1.6 kg BW chosen here, the GE contents of the piglets from the present study were also numerically higher (19.6 MJ GE/kg DM to 18.6 MJ GE/kg DM). This is probably due to the higher crude fat contents, as well as the consistently lower crude ash contents of the piglets in the present study.

The calcium and phosphorus contents essentially reflect the development of the skeleton. There are indications from the literature that the calcium content of the newborn in particular varies considerably in some cases. Whilst Widdowson [46] determined the calcium content to be 9 g/kg (on a fresh basis), there are also studies in which the calcium content assumed values of 13.8 g/kg (on a fresh basis) [47]. It is also reported that such fluctuations in phosphorus content do not occur [30]. This can be confirmed in the present study insofar as the standard deviations of the calcium contents were generally higher than those of the phosphorus contents. However, the fluctuations across all four weight classes were limited to a range of 9.16–11.7 g/kg (on a fresh basis). The reference values for phosphorus given in the literature [30] (5.0–6.8 g/kg on a fresh basis) can be fully confirmed by the results of the present study. The significantly higher calcium and phosphorus contents (in DM) of piglets with a BW of <0.8 kg at the time of birth are striking. This confirms the statement from Widdowson [46], who also found higher calcium and phosphorus contents in lighter piglets. The proportion of the skeleton determines predominately the inorganic fraction of the body of very light piglets, which thus appears to be relatively high. A similar correlation can also be found in the trace element contents. Here, particularly light piglets had significantly higher zinc contents (in DM), which may be due to their relatively larger skin surface area in relation to their body volume.

Both from the farmer’s economic point of view and due to veterinary indications, the trace element iron should be mentioned. Newborn piglets and puppies have the lowest iron levels at the time of birth when compared to other animal species [32]. According to Meyer and Kamphues [32], these are just 237 mg/kg DM, whereas foals, for example, have iron levels of 440 mg/kg DM. In the present study, however, depending on the BW at the time of birth, these varied between 136 (<0.8 kg BW) and 180 mg/kg DM (>1.6 kg BW). Iron is an essential trace element as it is involved in many key functions in the organism. One of these is heme or hemoglobin synthesis and oxygen transport via hemoglobin. According to a study by Miles et al. [48], a higher oxygen-binding capacity can, in turn, result in a higher vitality in piglets immediately postnatum. The piglet’s endogenous iron reserves are quickly depleted, and even the sow’s colostrum cannot compensate for this deficit, as it contains just around 2.84 µg iron/mL [49]. Common practice has therefore changed to administering approx. 200 mg iron parenterally as iron dextran on the 2nd or 3rd day postnatum [29]. There are also studies that can prove that the iron content in the liver of the newborn piglet is influenced by the iron content in the sow’s feed. In a study by Buffler et al. [50], pregnant sows were fed different iron concentrations of 114 mg/kg DM and 256 mg/kg DM in the compound feed. The daily feed intake was limited to 2.5 kg for both groups and to 3.2 kg from day 85 onwards. Newborn piglets from the sow group with 114 mg iron/kg DM in the compound feed had 40% lower iron contents in the liver (500 vs. 809 µg/g DM) when compared to piglets from the sow group with 256 mg iron/kg DM in the compound feed. It would therefore seem obvious to increase the sow’s iron supplementation in order to achieve higher iron levels in the newborn piglet. However, since the above-mentioned study involved litter sizes of 9.8 and 12.1 piglets, the question inevitably arises as to the iron transport capacity of the placenta, and, against the background of the decreasing uterine blood flow per fetus in large litters [51], whether this is not exhausted at some point with the increasing number of piglets.

The placental weight from the study used for comparison [31] was 2.93 kg on average. Accordingly, it was assumed that 19.8% placental weight was added per kg of newborn piglet (19.8% of 21.2 = 4.20 kg placental weight). Moreover, in the present study, the placenta from sows with litters > 16 piglets born were assumed to have a GE content of 1.60 MJ/kg of newborn piglets. Accordingly, the total placental weight contained 6.72 MJ GE. This means that the placental weight also contained significantly more GE (33.5%) than in the study by Beyer et al. [31].

To derive the requirement for gestation, the Society of Nutrition Physiology in Germany (GfE) is based on an energy content of 4.8 MJ per kg of conception products at the end of gestation [52]. The fetus accounts for 72%, the placenta for 12%, and the fluids and uterine weight gain for a further 16% [53]. If the GE of the total litter and placental weight from the studies by Beyer et al. [31] is totaled against this background, an absolute value of 53.5 MJ GE follows. If a further 16% is then added to take into account the fluids and uterine weight gain, the total energy estimate for the entire gestation period is 62.1 MJ. If this is calculated in the same way for the GE content of the piglets and placentas from the present study, or is based on the performance data of the sows from the studies by [54], a total energy requirement of 99.8 MJ is obtained for the entire gestation period. In absolute terms, this is therefore a considerable increase in performance on the part of the pregnant sow, even though the GE content per kg of piglet weight is 4.71 MJ and thus, as before, corresponds to the value stated by Noblet et al. [52].

Furthermore, the GfE [55] assumes an energy retention in the conception products of 1 MJ/day during the low gestation phase (1st–85th day) and 2.5 MJ/day during the late gestation phase (from 85th–115th day). This results in an energy retention in the conception products of 160 MJ over the entire gestation period. The energy retention in the mammary gland is additionally taken into account in the form of 1 MJ/day during the late gestation period. This would result in a total retention (conception products + mammary gland) of 190 MJ for the entire gestation period, which still seems to meet the requirements of today’s modern sow lines. The only uncertainty factor would be the formation of the udder and the accessibility for the piglets to functional teats, which is associated with piglet survival and must be considered, especially in sows in their first pregnancy.

The protein intake during pregnancy is calculated using the GfE [55], taking into account protein retention in the uterus and the mammary gland and the development of 12 or 13 fetuses with a total weight gain of 24–26 kg in total. An average protein retention of 250 g per piglet born is assumed for different litter sizes. Firstly, the proportion attributable to the mammary gland can be subtracted, so that the protein retention in the conception products including the uterus then amounts to 214 g per piglet born.

As no data on the energy and nutrient intake in the gravid uterus were collected in the present study, the proportion attributable to the uterus should also be neglected for the intended comparison with the protein intake of the pregnant sow. This could be based on the studies by Noblet et al. [52], who estimated the proportion of energy intake in the uterus to be 11%. This should be possible insofar as the energy content in the uterus is largely determined by the crude protein content [31]. After the deduction of 11%, a protein content per piglet with placenta of 190 g would remain. The average BW of a newborn piglet in the years 2000–2006 was about 1.4 kg; the estimated proportion of placenta for this is approx. 19%, according to the results of this study, i.e., 277 g. If this is now related to 1 kg of piglet weight (and its associated placenta), this would result in a protein retention of 136 g. In the present study, the highest crude protein content per kg on a fresh basis was found in piglets with a calf weight of 0.8–1.2 kg at birth, which totaled 123 g. In addition, there would be around 54 g crude protein/kg placenta or, in relation to 1 kg piglet weight, around 10–11 g crude protein. In total, this would result in about 135 g crude protein per kg of piglet weight (including placenta), so that the GfE’s recommendations [55] for protein, due to the already very high assumed litter weight (even though a lower number of piglets born is assumed), still guarantee a supply of high yielding sows, which is appropriate to their needs and performance in view of the increased reproductive performance of the sows. For this purpose, the results of the chemical analyses of the present study were compared with the results of Sagel [33] and Beyer et al. [31], were and put in relation to the data of the [55]. An increase in live weight of 25 kg was assumed due to the increase in the weight of conception products and mammary gland, as well as 13 fetuses, depending on parity [55].

## 5. Conclusions

The present study clearly shows that, when compared to earlier studies, the energy and protein accretion in the total fetal mass (and the whole placental mass) increased by about 75% (compared to Beyer et al. [31]: number of piglet = 10, litter masses = 13.8 kg and an average piglet weight = 1.3 kg but today: number of piglets = 16.3, litter masses = 21.2 kg with an average piglet weight = 1.3 kg at birth), essentially as a result of the larger fetal weight, and not as a result of a different body composition.

The results are plausible in that today, by all means, newborn piglets with a body weight of 1.0 kg scarcely have impaired chances of survival, which is reflected in their normal energy content. On the other hand, the energy and nutrient supply for high yielding sows at the end of the gestation must be significantly augmented, as the main influencing factors, namely energy and protein accretion, have increased considerably.

## Figures and Tables

**Table 1:** Chemical composition of newborn piglets according to body weight at the time of birth.

Parameter (g/kg Dry Matter)	Body Weight (kg)			
	<0.8 (*n* = 5)	0.8–1.2 (*n* = 5)	>1.2–1.6 (*n* = 10)	>1.6 (*n* = 5)
Dry matter (g/kg fresh basis)	183 ^b^ ± 14.2	200 ^a^ ± 6.83	191 ^ab^ ± 7.68	187 ^ab^ ± 13.6
Crude ash	232 ^a^ ± 29.0	187 ^b^ ± 4.69	187 ^b^ ± 14.2	185 ^b^ ± 1.92
Crude protein	646 ^a^ ± 39.9	617 ^ab^ ± 15.9	597 ^b^ ± 16.6	597 ^b^ ± 29.6
Crude fat	60.1 ^b^ ± 4.61	74.1 ^a^ ± 10.3	71.6 ^ab^ ± 17.0	69.4 ^ab^ ± 16.7
Nitrogen-free extract	61.7 ^b^ ± 35.7	122 ^a^ ± 23.2	145 ^a^ ± 25.9	148 ^a^ ± 44.4
Gross energy (MJ/kg DM)	18.9 ^b^ ± 0.709	19.8 ^a^ ± 0.365	19.6 ^a^ ± 0.593	19.6 ^a^ ± 0.579

^a,b^ Means in a row with different superscripts differ significantly (*p* < 0.05).

**Table 2:** Nutrient and energy contents of stillborn or a liveborn piglets (>1.2–1.6 kg BW) at birth.

Parameter (g/kg Dry Matter)	Stillborn Piglets (*n* = 3)	Liveborn Piglets (*n* = 7)
Dry matter (g/kg fresh basis)	187 ^a^ ± 6.66	193 ^a^ ± 7.80
Crude ash	189 ^a^ ± 8.89	186 ^a^ ± 16.6
Crude protein	591 ^a^ ± 18.0	599 ^a^ ± 16.7
Crude fat	60.9 ^a^ ± 1.17	76.2 ^a^ ± 18.7
Nitrogen-free extract	160 ^a^ ± 12.7	139 ^a^ ± 28.3
Gross energy (MJ/kg DM)	19.3 ^a^ ± 0.208	19.8 ^a^ ± 0.668

^a^ Means in a row with similar superscripts not differ significantly (*p* < 0.05).

**Table 3:** Mineral content of newborn piglets according to body weight at birth.

Parameter	Unit	Body Weight (kg)			
		<0.8 (*n* = 5)	0.8–1.2 (*n* = 5)	>1.2–1.6 (*n* = 10)	>1.6 (*n* = 5)
Calcium	g/kg DM	64.2 ^a^ ± 8.18	48.4 ^b^ ± 3.41	48.0 ^b^ ± 4.02	51.0 ^b^ ± 1.80
Magnesium		1.71 ^a^ ± 0.402	1.33 ^b^ ± 0.115	1.30 ^b^ ± 0.130	1.29 ^b^ ± 0.054
Phosphorus		37.3 ^a^ ± 4.61	29.7 ^b^ ± 1.99	29.1 ^b^ ± 1.78	30.1 ^b^ ± 1.22
Sodium		12.7 ^a^ ± 0.536	9.77 ^b^ ± 0.723	9.83 ^b^ ± 1.17	9.38 ^b^ ± 0.554
Potassium		9.78 ^a^ ± 0.794	8.18 ^b^ ± 1.18	8.28 ^b^ ± 0.706	8.30 ^b^ ± 0.411
Chloride		13.2 ^a^ ± 1.53	10.0 ^b^ ± 0.490	10.7 ^b^ ± 0.789	10.6 ^b^ ± 0.583
Sulfur		6.95 ^a^ ± 0.369	6.53 ^b^ ± 0.168	6.37 ^b^ ± 0.269	6.37 ^b^ ± 0.333
Copper	mg/kg DM	15.0 ^a^ ± 4.25	14.5 ^a^ ± 0.926	11.8 ^a^ ± 2.98	12.3 ^a^ ± 3.95
Zinc		90.3 ^a^ ± 12.2	83.4 ^a^ ± 1.67	74.3 ^b^ ± 11.2	74.2 ^b^ ± 12.9
Manganese		6.81 ^a^ ± 2.14	6.09 ^a^ ± 0.715	6.23 ^a^ ± 1.14	5.44 ^a^ ± 1.86
Selenium		0.475 ^a^ ± 0.073	0.454 ^a^ ± 0.055	0.402 ^a^ ± 0.148	0.503 ^a^ ± 0.111

^a,b^ Means in a row with different superscripts differ significantly (*p* < 0.05).

**Table 4:** Nutrient and energy content of placenta from the litters with the number of born piglets ≥14–16.

Parameter	Unit	Placenta
Dry matter (DM)	g/kg fresh basis	72.2 ± 8.13
Crude ash	g/kg DM	128 ± 11.5
Crude protein		742 ± 16.8
Crude fat		65.1 ± 3.58
Nitrogen-free extract		64.4 ± 14.4
Gross energy	MJ/kg DM	21.5 ± 0.281
Calcium	g/kg DM	7.10 ± 1.10
Magnesium		1.07 ± 0.118
Phosphorus		7.90 ± 0.731
Sodium		32.3 ± 4.69
Potassium		10.0 ± 1.28
Chloride		42.1 ± 6.42
Sulfur		9.43 ± 0.499
Copper	mg/kg DM	12.8 ± 4.24
Zinc		55.0 ± 5.64
Iron		367 ± 60.5
Manganese		6.80 ± 2.33
Selenium		1.23 ± 0.171

## Data Availability

The data presented in this study are available in this manuscript.

## Supplementary Materials

The following supporting information can be downloaded at https://www.mdpi.com/article/10.3390/ani14091380/s1, Table S1: Amino acid contents of newborn piglets according to body weight at the time of birth.
