# Supplementary material for: Chemical Composition of Newborn Piglets with Different Weights at Birth in Sows with a High Reproductive Performance
Source: Animals (Basel). 2024 May 4;14(9):1380. doi: 10.3390/ani14091380 (PMC11082942; doi:10.3390/ani14091380)
Supplement: Supplementary file 1 [file animals-14-01380-s001.zip › animals-2991137-supplementary.pdf]

Supplementary file

Table S1. Amino acid contents of newborn piglets according to body weight at the time of birth.

**Parameter (g/kg DM)**	**Body weight (kg)**						
	**<0.8 (*n*=2)**		**0.8-1.2 (*n*=2)**		**>1.2-1.6 (*n*=4)**	**>1.6 (*n*=2)**	
Aspargine	54.2	49.8	50.0	52.4	49.0±1.33	47.6	50.7
Threonine	24.7	21.8	20.1	22.4	22.0±1.05	18.8	22.6
Serine	31.0	27.5	27.6	30.1	28.8±2.11	25.3	28.9
Glutamine	91.1	82.5	80.8	86.3	83.3±2.88	76.5	85.0
Glycine	66.0	62.7	65.6	66.3	61.3±3.26	62.6	64.0
Alanine	44.0	41.5	42.5	43.0	41.1±1.89	40.8	42.3
Valine	33.2	29.2	28.3	30.6	28.2±0.850	27.7	30.0
Cysteine	5.65	5.26	4.86	5,79	7.91±1.32	4.59	5.31
Methionine	8.15	8.59	7.67	8.15	8.90±0.822	7.69	8.42
Ileucine	19.4	18.2	16.5	17.7	18.1±0.623	15.8	17.5
Leucine	46.1	40.9	41.5	43,7	42.7±1.50	39.4	42.8
Tyrosine	18.2	16.1	15.9	16.9	16.6±0.873	15.0	17.7
Phenyalanine	24.4	22.0	21.8	23.0	25.2±1.16	20.8	22.5
Histadine	14.8	12.8	14.5	14.4	14.5±0.625	13.9	14.3
Lysine	41.1	36.2	37.5	38.3	38.8±2.27	35.3	38.1
Arginine	43.5	40.8	40.7	42.4	42.0±5.24	38.2	40.7
Proline	45.4	40.7	41.3	45.1	44.5±3.17	39.8	44.2
